# A New Generation Microarray for the Simultaneous Detection and Identification of *Yersinia pestis* and *Bacillus anthracis* in Food

**DOI:** 10.1155/2012/627036

**Published:** 2012-10-18

**Authors:** Noriko Goji, Trevor MacMillan, Kingsley Kwaku Amoako

**Affiliations:** ^1^Canadian Food Inspection Agency, National Centres for Animal Disease, Lethbridge Laboratory, P.O. Box 640, Lethbridge, AB, Canada T1J 3Z4; ^2^Department of Biological Sciences, D872 University Hall, 4401 University Drive, University of Lethbridge, Lethbridge, AB, Canada T1K 3M4

## Abstract

The use of microarrays as a multiple analytic system has generated increased interest and provided a powerful analytical tool for the simultaneous detection of pathogens in a single experiment. A wide array of applications for this technology has been reported. A low density oligonucleotide microarray was generated from the genetic sequences of *Y. pestis* and *B. anthracis* and used to fabricate a microarray chip. The new generation chip, consisting of 2,240 spots in 4 quadrants with the capability of stripping/rehybridization, was designated as “Y-PESTIS/B-ANTHRACIS 4x2K Array.” The chip was tested for specificity using DNA from a panel of bacteria that may be potentially present in food. In all, 37 unique *Y. pestis*-specific and 83 *B. anthracis*-specific probes were identified. The microarray assay distinguished *Y. pestis* and *B. anthracis* from the other bacterial species tested and correctly identified the *Y. pestis*-specific oligonucleotide probes using DNA extracted from experimentally inoculated milk samples. Using a whole genome amplification method, the assay was able to detect as low as 1 ng genomic DNA as the start sample. The results suggest that oligonucleotide microarray can specifically detect and identify *Y. pestis* and *B. anthracis* and may be a potentially useful diagnostic tool for detecting and confirming the organisms in food during a bioterrorism event.

## 1. Introduction

Microarray technology has great potential for use in diagnostics, and DNA microarrays have received considerable attention due to the ability to simultaneously analyse a very large number of nucleic acid sequence targets and detect multiple genetic targets or genomes from multiple pathogens on a single slide [1]. The technology has played an increasingly important role in genomics and has generated increased interest in the last decade. 

DNA microarrays consist of several oligonucleotide probes that have been immobilized on a solid glass support, and the technique has great potential to be used for the discrimination of closely related strains by employing oligonucleotides specific for each target organism. Hence, the design of a suitable probe set is the key in the development of microarrays as all probes on a microarray should be highly specific for their target genes. The probes should be able to bind efficiently to target sequences to allow the detection of very low abundance targets in complex mixtures with high sensitivity [2]. The use of DNA microarrays has been shown to be effective for the high-throughput detection of pathogenic microorganisms in clinical, environmental, food, and water samples [3–8]. However, the application for the detection of biothreat agents in food has not been documented. Food is considered a vulnerable target for bioterrorist attack, and events related to the deliberate contamination of food using conventional foodborne pathogens such as *Salmonella* [9] make foodborne bioterrorism involving *Y. pestis* and *B. anthracis* a possibility. Foodborne bioterrorism response preparedness involving *Y. pestis* and *B. anthracis* is required to deal with any potential threat involving the food supply. The development of a species-specific method for simultaneous detection of biothreat agents from food is essential. 

In this study, we describe an approach that involves the use of a new generation microarray to allow the simultaneous detection and identification of *Y. pestis* and* B. anthracis* from food. We designed and tested probes based on the virulence genes from the two biothreat agents and demonstrate that this microarray approach has the potential to be used for the specific detection of *B. anthracis* and *Y. pestis* in a foodborne application.

## 2. Materials and Methods

### 2.1. Microarray Probe Design and Chip Fabrication

Whole genome sequences of *B. anthracis* Ames [10] and *Y. pestis* CO92 [11] from GenBank were selected for the custom design of around 35-mer probes and used to fabricate a low density custom array. The oligonucleotide probes were generated from the virulence plasmids, and a 4x2K chip array (four identical arrays of 2,000+ spots), designated as “Y-PESTIS/B-ANTHRACIS 4x2K Array,” was custom-designed (CustomArray Inc. USA, formerly CombiMatrix). In all, 533 and 1,707 probes were generated for *Y. pestis* and *B. anthracis*, respectively. With this 4x2K chip design, eight identical experiments can be run on a single chip. The oligonucleotide probes are electrochemically synthesised directly onto the chip and does not require the need to order them separately for resuspension and spotting using a robot. The above described features are lacking in the design of the old microarray chips and make this a new generation microarray. Another unique feature of this new generation chip is that it can be stripped and reused for up to three times. Each CustomArray 4x2K microarrays can be stored in a cool dry place for up to 4 months.

### 2.2. Bacterial Strains and DNA Extraction

The *B. anthracis *strains used in this study were kindly provided by Dr. Elizabeth Golsteyn Thomas (Canadian Food Inspection Agency) and Mr. Doug Bader (Defence Research Development Canada). The sources of all the other bacterial strains used are provided in a previous paper [12] and listed in Table 1. Bacteria were grown on Tryptic Soy Agar (Becton, Dickinson and Company, USA) supplemented with 5% sheep blood, and single colonies were transferred subsequently into Tryptic Soy Broth at 37°C overnight. Genomic DNA was extracted as previously described [12].

### 2.3. DNA Amplification and Labeling

**Figure 1:**
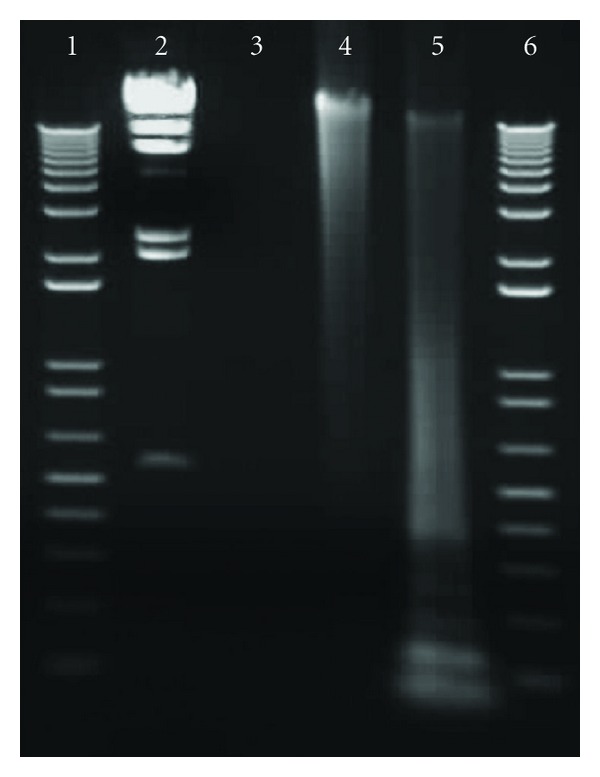
1% agarose gel loaded with *B. anthracis* DNA before and after amplification using REPLI-g genomic DNA amplification kit and *RsaI* digestion. Lanes 1 and 6, :  1 kb molecular weight markers, lane 2: Hind III molecular weight marker (23130, 9416, 6557, 4361, 2322, 2027, and 546 bp), lane 3, : 5 *μ*L of 20 ng/*μ*L *B. anthracis* Ba 179 genomic DNA, lane 4, : 5 *μ*L of REPLI-g amplified DNA (355 ng/*μ*L), and lane 5: *RsaI* digested DNA.

Genomic DNA was amplified using REPLI-g Mini Kit (Qiagen GmBH, Hilden, Germany) (Figure 1), and amplified DNA provided consistent hybridization results (data not shown). For labeling, 2 *μ*g of amplified DNA was digested with *RsaI* (Life Technologies, Carlsbad, CA, USA) at 37°C for 4 hours and subsequently labeled with Alexa Fluor 555 and/or 647 florescent dyes using BioPrime Plus Array CGH Genomic Labeling Kit (Life Technologies, Carlsbad, CA, USA) following the manufacturer's instructions. Concentration and labeling efficiency of DNA was determined using the NanoDrop ND-1000 fluorometer (Thermo Fisher Scientific, Waltham, MA, USA). Labeled DNA was stored at −20°C in amber microcentrifuge tubes unless used for hybridization immediately. For specificity studies, genomic DNA of *Y. pestis* CO92 or a wild-type *B. anthracis* isolate number 179 was mixed with the genomic DNAs extracted from a panel of bacteria listed in Table 1 at different ratios (1 : 1, 1 : 3, 1 : 9, and 1 : 19 in weight). DNA amplification and labeling were done as described above.

### 2.4. DNA Hybridization to Microarray and Chip Stripping

Hybridization of labeled genomic DNA to the CustomArray 4x2K format slide was performed according to the manufacturer's protocol (CustomArray Inc. Bothell, WA, USA). Each microarray consisting of 4 identical array sectors was individually loaded with different DNA samples. By using two florescent dyes, eight samples were tested simultaneously on one microarray slide. Briefly, 30 *μ*L of hybridization solution containing 100 ng of two fluorochrom-labeled (Alexa Fluor 555 or 647) DNA was pipetted into each of the four chambers and covered with foil adhesive tape to avoid light. The covered slide was incubated in a humid hybridization rotisserie oven (UVP, LLC, Upland, CA, USA) for 16 hours at 50°C with gentle rotation. All labeled DNA samples were hybridized in duplicate with either Alexa Fluor 555 or 647 to ensure consistency of the results.

Following hybridization and imaging, microarrays were submerged in 0.5 M sodium hydroxide at room temperature for 15 minutes and stripped using the CombiMatrix CustomArray Stripping Kit (CustomArray Inc.). Stripped slides were scanned before rehybridization to make sure there were no more signals and stored in a slide holder containing PBS at 4°C. Rehybridization for a single slide was repeated a maximum of two times.

### 2.5. Microarray Data Analysis

Hybridized microarrays were imaged using the Axon 4000B Microarray Scanner (Axon Instruments, Molecular Devices, LLC, Sunnyvale, CA, USA). Hybridization of each sample was done in duplicate, and scanning was done in triplicate. The TIFF images were analyzed using the GenePix Pro software Version 5.0 (Axon Instruments), and the data was extracted for further analysis of the total intensity of each spot. All data were transferred to Microsoft Excel for Cluster and TreeView analysis (Stanford University, CA, USA), and then heat maps were generated following the instructions of the software [13]. 

### 2.6. Application of Microarray for the Analysis of Spiked Food Samples

To investigate the use of the microarray for a foodborne application, *Yersinia pestis* strain Pp1967 was cultured overnight in TSB at 28°C, and about 10^6^ CFU/mL was inoculated into 25 mL of 1% skimmed milk purchased from a local grocery store. A 225 mL volume of buffered peptone water was added, and the spiked milk sample was stomached for 2 minutes using a stomacher (Seward Ltd., West Sussex, UK). The samples were aliquoted into 10 mL volumes in 15 mL of Falcon tubes, boiled for 10 minutes to kill cells, and centrifuged. Pellets were collected, and DNA was extracted using the DNeasy Blood and Tissue Kit (Qiagen GmBH, Germany). The extracted DNA was labeled, amplified, digested and hybridized to the chip as described above.

## 3. Results 

### 3.1. Hybridization of Genomic DNA from *Yersinia pestis *and* Bacillus anthracis* Strains

Six *Y. pestis* strains and two *Y. pseudotuberculosis* strains were examined to evaluate the positive spots. All DNA samples were tested in duplicate, and the total fluorescent intensity (TFI) data for all 2,240 probes were assembled after subtracting the average intensity of ten negative spots. Spots showing abnormality on the microarray slides due to hybridization failures were filtered out. The TFI values higher than 20,000 were selected as positive signals. This number was determined based on the results from normalized data from *Y. pestis* CO92 or *B. anthracis *Ames, showing 0 (the median) of log-transformed (log_2_) values. For *B. anthracis,* the Ames strain and 7 wild-type isolates from animals in Canada from 1996 to 2006 and 4 *Bacillus* species (*B. cereus, B. thuringiensis, B. *coagulans, and* B. subtilis*) were also examined (Table 1). The data obtained from genomic DNA cocktails of either *Yersinia* spp. or *Bacillus* spp. and foodborne pathogens were analyzed against positive probes of the two bacterial agents. About 300 probes were found to be positive for each *Y. pestis *strain and out of this, 72 of them gave positive values in three or more strains. Subsequently, positive probes from *Y. pseudotuberculosis* ATCC 29833 and Turku strains, and genomic DNA cocktails of *Yersinia spp.* or foodborne pathogens were compared to the 72 probes, and finally 37 were selected as *Y. pestis*-specific probes. For *B. anthracis*, about 800 targets were analyzed and after analyzing the positive probes from the DNA cocktails of 4 *Bacillus* species and foodborne pathogens, 83 were selected as *B*. *anthracis*-specific.

The sequences of the 37 *Y. pestis*-specific probes are shown in Table  2a and the 83 probe sequences for *B. anthracis* in Table  2b (see Supplementary Material available online doi:10.1155/2012/627036). 

### 3.2. Specificity and Sensitivity of the Specific Probes for *Y. pestis* and *B. anthracis*

To examine the specificity of the 37 specific probes for *Y. pestis* and 83 for *B. anthracis*, genomic DNA from the two organisms was mixed with those from a panel of foodborne bacterial pathogens and subsequently amplified with REPLI-g before loading onto the microarray slides. This was to mimic the detection of *Y. pestis* and *B. anthracis* from food samples if contaminated with other pathogens. The results showed that all the probes had strong positive signals (data not shown). 

**Figure 2:**
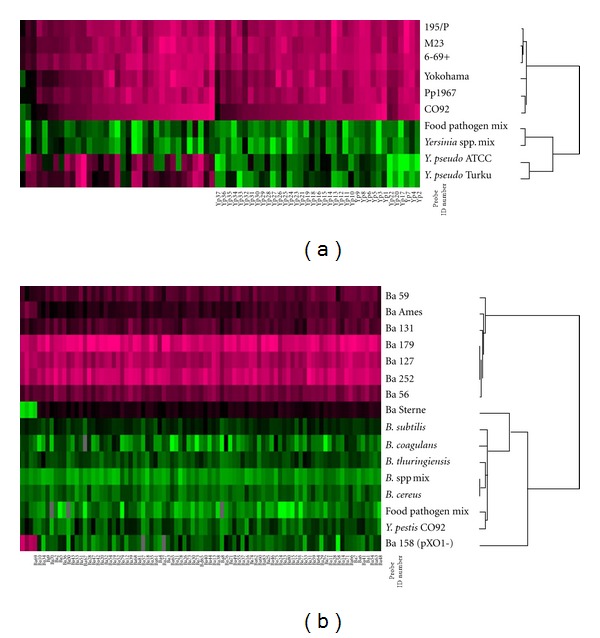
Unique patterns of the heat map and clustering images generated from the data of *Y. pestis* (a) and *B. anthracis *(b) specific probes. The total intensity of the positive spots for either *Y. pestis* or *B. anthracis* was normalized using microarray data analysis software (Cluster and Treeview [5]) after converting to log_2_ scale. Probes with significant intensity are shown in pink. The 37 probes for *Y. pestis* and 83 for *B. anthracis* have probe ID numbers underneath the heat maps.

Figures 2(a) and 2(b) show the heat map and clustering results for *Y. pestis *and* B. anthracis. *The heat maps show unique patterns to each bacterial species and are easily distinguished from closely related organisms. Additionally, for *Y. pestis*, another 35 probes shown on the heat map with positive intensity levels in *Y. pseudotuberculosis* as well could be potentially considered as* Y. pestis-*specific probes since the signals were significantly higher than the ones from *Y. pseudotuberculosis.*

### 3.3. Milk Spiked with *Y.******* *pestis*

**Figure 3:**
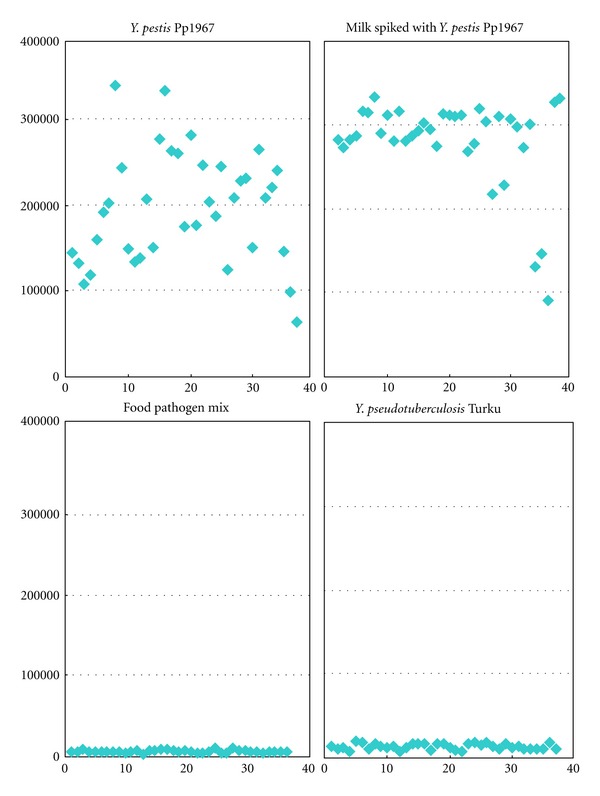
Total fluorescent intensity (TFI) of 37 *Y. pestis*-specific probes for amplified genomic DNA extracted from spiked milk samples. The *X*-axis numbers represent *Y. pestis* probe ID number. The *Y*-axis shows averaged TFI for each probe.

To demonstrate the direct application of this CustomArray DNA hybridization technique for detection in food, we extracted DNA from milk samples spiked with 10^6^ CFU/mL *Y. pestis* Pp1967. Strong signal intensities were shown in all 37 probe spots (Figure 3). These spots did not show any signal intensity in negative control milk samples indicating that the probes were specific for *Y. pestis*.

## 4. Discussion

Detection and confirmation of biothreat agents such as *Yersinia pestis* and *Bacillus anthracis* in food are very important for the effective protection of the public from any potential foodborne bioterrorism threat. To date, the simultaneous detection of these two biothreat agents in food using microarray such as described in this study has not been reported. Traditional methods used for the confirmation of pathogens such as enrichment culture, microscopy, serology, and biochemical assays have several limitations, in which they are laborious and time consuming and hence inefficient in addressing potential foodborne bioterrorism concerns. 

Rapid and specific identification of foodborne pathogens and biothreat agents is the key for early detection and quick response in the event of foodborne disease outbreak. Real-time PCR has recently been developed as a rapid and specific method for detecting biothreat agents in food [12, 14, 15]. Even though multiplex real-time PCR assay can amplify several different target regions of genomic DNA in a single reaction, there is limited information available from this technique; thus, a combination of different methods should be considered as an alternative. 

DNA microarray technology has been applied to foodborne pathogen detection [16, 17]. This approach has a strong potential not only to identify multiple pathogens with very high specificity in a single experiment, but also to be able to demonstrate genetic differences and similarities among bacterial strains [18, 19] and provide further characterization of bacterial isolates. To obviate the use of PCR which requires the design of specific primers and optimization of the reaction conditions, we amplified genomic DNA using QIAGEN REPLI-g whole genome amplification system. REPLI-g technology has been successfully used to amplify genomic DNA [20] and according to the report, the intensity values of REPLI-g amplified DNA can provide relevant information about copy numbers of the gene targets in the genome as amplification is uniform. The report showed that the higher the intensity values, the higher the copy number of the gene target. The high intensities observed with targets on plasmid pPCP1 in the present work confirm that this plasmid has a high copy number. The sensitivity of DNA microarrays is usually poor when total genomic DNA is used for hybridization [21]; however, this can be enhanced to allow detection of low level concentration of pathogens using labeling and hybridization of amplified products [2, 5, 22, 23]. The application of a random whole genome amplification step prior to labeling in the current study enabled the use of as low as 1 ng of DNA as the start material for the microarray analysis. This led to the successful simultaneous detection and identification of *Y. pestis* and *B. anthracis* in a single assay. This did not require the design of PCR primers to generate materials for labeling and hybridization. Furthermore, the stripping and reuse of the microarray chip including the high-throughput ability to run 4 separate experiments simultaneously on a single chip offers a unique new generation microarray and allows experiments to be replicated under identical conditions, thus ensuring high reproducibility of results. This is not the case with the use of the conventional microarrays where a chip is used once, and replication of experiments is done using different chips. 

Several reports have demonstrated the use of DNA microarrays for the detection of pathogens in food [24–27]. The technology has also been developed for biodefence applications involving *B. anthracis* and *Y. pestis* [28–30]. However, to our knowledge, the use of the technology for the simultaneous detection and identification of biothreat agents in food has not been documented. Here we demonstrate the first successful application in food biodefence involving the detection of *Y. pestis *in spiked milk samples. 

The high specificity of the species-specific probes is demonstrated by the lack of cross-reactivity with a panel of closely related and distantly related strains that may be potentially present in food. The 37 and 83 species-specific probes generated for *Y. pestis* and *B. anthracis*, respectively, should enable the use of microarray for the direct detection and identification from contaminated food without the need for isolating the biothreat agent. However, this number can be expanded to about 300 and 800 probes for *Y. pestis* and *B. anthracis*, respectively, and used for the confirmation of the bacteria if isolated in pure culture from contaminated food. The careful analysis of genetic sequence information and selection of probes contributed to this. In particular, the targeting of virulence genes from the virulence plasmids that are unique to the two organisms contributed to the high specificity observed. Previous studies involving the use of virulence genes in the design of microarrays for the detection of pathogenic bacteria have been reported [31–33]. The use of several virulence genes as detection markers offers more information on the virulence potential of strains implicated and adds more value to the use of microarray as a platform for the detection and confirmation of biothreat agents. 

In conclusion, the new generation microarray developed in this study is novel for the simultaneous detection and identification of *Y. pestis* and *B. anthracis* in food, and demonstrates the usefulness of microarray as a potential diagnostic tool for biodefence applications.

## Supplementary Material

Table 2a. *Yersinia pestis* specific probes.
Table 2b. *B. anthracis* specific probes.

## Figures and Tables

**Table 1:** Strains used in this study.

Species	Strain	Remarks
Foodborne pathogen mix		

*Proteus vulgaris*	ATCC 8427	
*Klebsiella pneumoniae*	ATCC 13883	
*Shigella dysenteriae*	ATCC 11835	
*Escherichia coli *O157H7	EDL933	
*Mannheimia haemolytica*	Z13	
*Vibrio vulnificus*	Z86	
*Citrobacter braakii*	ATCC 12012	
*Salmonella typhimurium*	71-471	
*Aeromonas hydrophila*	Z22	
*Pseudomonas aeruginosa*	ATCC 27853	
*Listeria monocytogenes*	ATCC 15313	
*Streptococcus pyogenes*	ATCC 19615	
*Enterococcus faecalis*	ATCC 29212	
*Micrococcus lysodeikticus*	Z9	
*Staphylococcus aureus*	Z13	
*Campylobacter jejuni*	ATCC 11168	

*Yersinia* spp. mix		

*Yersinia pseudotuberculosis*	ATCC 29833	
*Yersinia pseudotuberculosis*	Turku	
*Yersinia enterocolitica*	ATCC 23715	
*Yersinia kristensenii*	ATCC 33638	
*Yersinia frederiksenii*	ATCC 33641	
*Yersinia intermedia*	ATCC 29909	

*Bacillus* spp. mix		

*Bacillus cereus *	ATCC 14579	
*Bacillus subtilis*	NWBL 0060	
*Bacillus thuringiensis*	ATCC 10792	
*Bacillus coagulans*	ATCC 7050	

*Yersinia pestis* strains		

*Yersinia pestis*	Pp 1967	Wild-type isolate
*Yersinia pestis*	195/P	Wild-type isolate
*Yersinia pestis*	6/69H+	Wild-type isolate
*Yersinia pestis*	M23	Wild-type isolate
*Yersinia pestis*	Yokohama	Wild-type isolate
*Yersinia pestis*	CO92	NCBI Genome Ref Seq: NC_003143.1 [11]

*Bacillus anthracis* strains		

*Bacillus anthracis*	Ames	NCBI Genome Ref Seq: NC_003997.3 [10]
*Bacillus anthracis*	Ba 44	isolate from moose, NWT Canada in Aug 1993
*Bacillus anthracis*	Ba 56	isolate from cattle, ON Canada in Aug 1996
*Bacillus anthracis*	Ba 79	isolate from bear, NWT Canada in Jul 2000
*Bacillus anthracis*	Ba 127	isolate from caprine, BC Canada in Dec 2001
*Bacillus anthracis*	Ba 131	isolate from soil, Canada, date N/A
*Bacillus anthracis*	Ba 252	isolate from cattle, SK Canada in Jul 2006
*Bacillus anthracis*	Ba 59	isolate from bison, MB Canada in Jul 1998 Cap-
*Bacillus anthracis*	Ba 158	ATCC 4229 Tox–(pXO1-)
*Bacillus anthracis*	Sterne	NCBI Genome Ref Seq: NC_005945.1(pXO2-)
