# Supplementary material for: A New Generation Microarray for the Simultaneous Detection and Identification of Yersinia pestis and Bacillus anthracis in Food
Source: J Pathog. 2012 Oct 18;2012:627036. doi: 10.1155/2012/627036 (PMC3483683; doi:10.1155/2012/627036)
Supplement: Supplementary file 1 — Table 2a. Yersinia pestis specific probes. Table 2b. B. anthracis specific probes. [file 627036.f1.docx]

**Table 2a.** *Yersinia pestis* specific probes **(supplementary)**.

| **Probe ID** | **Sequence** | **Description** |
| --- | --- | --- |
| Yp1 | TTCATTGTCTCATGGCATCTATGGGAAACAGAC | ***pst*: pesticin**  Context: pPCP1-[4815-5888] |
| Yp2 | TTAAATACCTTTGGCAATTCATTGTCTCATGGCAT |  |
| Yp3 | ACCGTGTCATGGTTCTTGAGGGAACGA |  |
| Yp4 | TCCGATTGATACGGAGATGGAGAAAGACAGTG |  |
| Yp5 | TTGACCACGATATTTTCTCCAGTAATGGTTTTGAA |  |
| Yp6 | TCAATGACGCTAATATCGGTCTTCGGTTCAGC |  |
| Yp7 | TGCAGATAGACTTGATGGGTATTATATGCTTCGAG |  |
| Yp8 | TCGTTCGGTAAATATTGTTGACCACGATATTTTCT |  |
| Yp9 | CTGAAGGAATTTTTACGTCGTTCGGTAAATATTGT |  |
| Yp10 | GGTATTATATGCTTCGAGGGAAGGAGGCTTATGA |  |
| Yp11 | TCTTGAGGGAACGATTGCCGAATCTATTGAAC |  |
| Yp12 | GGTGTCCCTCAGTATATTGCAGATAGACTTGATGG |  |
| Yp13 | AATAACCGAAATCGCATCGTCCTAGCGGA |  |
| Yp14 | GTCATTTTCCGTCTAATCCTAGTAGCGATTATTTT |  |
| Yp15 | TCAAAAACTGAAGGGACCGTGTCTTACGAAC |  |
| Yp16 | ATGTCAGATACAATGGTAGTGAATGGTTCAGGTGG |  |
| Yp17 | TCTGTCTCTATTGGCGGAGATGCTGCC | ***pla***: **plasminogen activator protease**  **precursor**  Context: pPCP1+[6665-7603] |
| Yp18 | TTAACGCTGGATATTATGTCACACCTAATGCCAAA |  |
| Yp19 | AGGTACTCAGACCATTGATAAGAATAGTGGAGATT |  |
| Yp20 | AAACACGTTTCAGTTGGACAGCTACAGGTG |  |
| Yp21 | GGACTTGCAGGCCAGTATCGCATTAATGA |  |
| Yp22 | GAGTGCTAATGCAGCATCATCTCAGTTAATACCA |  |
| Yp23 | ATTATAAAGCAGGTATAACAGCAGGATATCAGGAA |  |
| Yp24 | AAACTTCCCGAAAGGAGTGCGGGTAATAGG |  |
| Yp25 | AATATATCCCCTGACAGCTTTACAGTTGCAGC |  |
| Yp26 | TTACATACAGTAAATATGATGAGGGCAAAGGAGGT |  |
| Yp27 | ACTATTCTGTCCGGGAGTGCTAATGCAGC |  |
| Yp28 | GGTATTTCCAATAAAAATTATACTGTGACGGCGGG |  |
| Yp29 | ATTCTTGAGGTCGGCTTGCAGATGATGC | **putative transcriptional regulator**  Context: pPCP1-[7790-8089]  similar to predicted transcriptional regulator  TR:P95258 (EMBL:Z84498) |
| Yp30 | TCACAAAAACAAGTTGCTGAGGCGATGGG |  |
| Yp31 | ATGGCAGATGAGATGATTCTTGAGGTCGGC |  |
| Yp32 | AAATGATTTAAAGCTGGCGACGTTAAAGCGTTAC |  |
| Yp33 | CGACGTTAAAGCGTTACGTTGAAGCAATGG |  |
| Yp34 | ATGAGAACATTAGATGAGGTGATTGCCAGTCGT |  |
| Yp35 | CCGATTCGTCGGGCTATCGTTCTTTGTG | **hypothetical protein**  Context: pPCP1-[8089-8436] |
| Yp36 | TCCGATAAGAGCCTTTTATGCGTTCGATCCG |  |
| Yp37 | GGCTGATTTGAAAAAGCTACAGGTTTACGGACC |  |

**Table 2b.** *B. anthracis* specific probes **(supplementary)**.

| **Probe ID** | **Sequence** | **Description** |
| --- | --- | --- |
| Ba1 | AATTTGTATCCTGTTTTCACATTTGGGCATTACGA | **acetyltransferase**; gnat family; (pxo1-37) |
| Ba2 | AGAGGAAATTCCGTTCTTCTTCTGTAAACCCAGC | **bacterial type ii/iv secretion system protein**  (pxo1-59) |
| Ba3 | CTCCTGTGGCACTTATTTCAATTGTTCTTCTTGC | **caax amino terminal protease family protein** (pxo1-85) |
| Ba4 | GGCTAAAAATGTAGAAGGGGTCTTGAAGCCG | **calmodulin-sensitive adenylate cyclase** Context:pXO1+[122608-125010] |
| Ba5 | TACAAGCACATAAGATCCCAGCAAGCTCGG | **conjugation protein; trag/trad family**  (pxo1-42) |
| Ba6 | GCAGAGCAACTACAAATACTTGTTAAACTGTTACA | **DNA topoisomerase I**  Context:pXO1-[176759-179371] |
| Ba7 | TAACAGATTCCCTAAGTCAACAACTGGAGGTAAAA | **group ii intron reverse transcriptase/maturase**  (pxo1-23) |
| Ba8 | GCCTACCGCAATATTAAGGGGAACAAAGGAA |  |
| Ba9 | TAAAGAAATTTCGAAGCCCAACGGACAAACCA |  |
| Ba10 | CCTGTCATTATTGTAAAAAGGCTGCACCAGATATC | **hypothetical protein**  Context:pXO1 |
| Ba11 | CCAAAACGCTATATTGAAATTGAGTTACCACATGG |  |
| Ba12 | AAGGATTCAAACGTAGGACGTATCCCTTTAAGTAA |  |
| Ba13 | GCCTCTATTGACAAGTTAAAGTTACGAGGACAACG |  |
| Ba14 | GAGAGCTTTCTAGGGAGAACCCAGCTAAAATGG |  |
| Ba15 | ACCCTAATGCATATGAATTTGATTATAATGGCGCT |  |
| Ba16 | CAATCCGTTACTCGCGCTAGAAAACAAATTATGTT |  |
| Ba17 | GGCCAAAACTTTAATGGTGAAGCAATAAGGAGAAT |  |
| Ba18 | TGCAACTGAATAGAGGATTAAATGCACATGGAGT |  |
| Ba19 | TGAAGCAATAAGGAGAATGCAACTGAATAGAGGAT |  |
| Ba20 | TTAAATGCACATGGAGTTCTAGGGCAAGAATTTGA |  |
| Ba21 | AATGATTACCCACAAGGTATATCGCCAGGTGA |  |
| Ba22 | TAACCGTAGATTGAAACCGAGAGAACATGAGGA |  |
| Ba23 | CGGATTACGAACTCGAAAAGTATTACAAAGGAAAA |  |
| Ba24 | AACCATATCAAATTGGCTGAGAGAGGCTTATATAG |  |
| Ba25 | AGCAAAAACAGCGTGAAACCATATCAAATTGGC |  |
| Ba26 | AGTGGGGAATTGAATCATCGACATTACGTAAAAGG |  |
| Ba27 | ATTGGCACGACATATGCTGTGACTCGATTT |  |
| Ba28 | TGGTCCGCTAGAAAAAGCATTAGAAGCCATG |  |
| Ba29 | ATTCTGTCTATTGAGACGGGGTATTCAAAACACG |  |
| Ba30 | CATATTGTATATAGATTTGGTCCGCTAGAAAAAGC |  |
| Ba31 | TCATTAGCATTAGGATTCGTAGAGATGGTGATAAA |  |
| Ba32 | CCAACTTTGGCGTCATTGAAAAACCAGTCAA |  |
| Ba33 | CGTAATAGGTCTATCTATCTATTTTTCCTCTGTTG |  |
| Ba34 | ACTTTTAGATAGAGGATTATTGCCTTTTGGCGTAG |  |
| Ba35 | ACTCACATGAACCCTTTCCCTACTGCAATTATTT |  |
| Ba36 | CACTTACCTATCGACAATAAATACTGAGGATCTTA |  |
| Ba37 | CATCGTTTTTAGGACATGTTATGCACCCATCTATT |  |
| Ba38 | GGAAAGATTTCGTAGAGTCAGGAAGAGATGTTATT |  |
| Ba39 | CATCTGCGTTATGCACCATCGTTTTTAGGACA |  |
| Ba40 | GTGCGCTATGAAAAGAAACAAGTCCAGGTATTACG |  |
| Ba41 | GGCGTAATGATTTAAAACATGCAAGTGATGTGATG |  |
| Ba42 | CTGTTCAACAAAGAGTAGCTGATGTTAAAGATATG |  |
| Ba43 | ATGTACCAAATTCGTGGGTCAGTTCACTTAGTATG |  |
| Ba44 | CCAATTAAGAGGATTCGTGCAATTAAAGGTGAAAT |  |
| Ba45 | CAGAACATTACATCAACAGAGAATCAGAAATGCAT |  |
| Ba46 | ATATTTTTCGTCATCCCAACTCAGCTAGATACAAT |  |
| Ba47 | TGGATTTGAGAAAGTTTTTAAAGAGGTTCAAGAGG |  |
| Ba48 | CCGCGAAGTATTGTTAAAACGAAAAGATTGGCT |  |
| Ba49 | AAGAGAACCTTCTACTAAGTAATTGCTGGAATAAC |  |
| Ba50 | TTCCAAAAGGATGATTGAAGAGAACCTTCTACTAA |  |
| Ba51 | GGCTACAAGAAGTTTGCTGAAGACATTTTATCTGC |  |
| Ba52 | ATCAGATGTTTAATGAAGCGAAGCCAATGATGTTT |  |
| Ba53 | AGATCCATTGAATTCACACATACACCAGCAACT |  |
| Ba54 | ATAAAGACGCAATATGGTATGGGTCAGTAAACTAT |  |
| Ba55 | AGTATTGGCCCCTTTAATCATGAAAACCCCAT |  |
| Ba56 | TTAATTTCGCACCTTATAATGGATGATACTGAAGC |  |
| Ba57 | TATTGATACCGTTAGTAGGTGAAAAAGTGGTGCG |  |
| Ba58 | TGCAGAAGAATATCGATATATGGAGTTTAAGGAGG |  |
| Ba59 | TCGTCTTTTTGCTATTACGCCTTCAATTAATGGAG |  |
| Ba60 | TTGACGACCCTTTTATTGAGGTTGCTGAGG |  |
| Ba61 | AAAAATATATGGAAGTCTCAATGGCTCTGAATGTG |  |
| Ba62 | CGTTATGACGGAAAAGTATGGACTTGTAGGACAAA |  |
| Ba63 | AAACATCAGTGATTCCAAGCTCTTCAATCGCT | **is231-related; transposase**  (pxo1-35) |
| Ba64 | TAGCTATTATCTGTTTCTGGATTAGTCAACATGTC |  |
| Ba65 | CAACATCTTACTCCCTCATTTTTAGAGAATCTGGC |  |
| Ba66 | TGAAAATTGGCGATCATTAGTAGATCCTGGTGATG | **protective antigen-related protein**  (pxo1-111) |
| Ba67 | ACGCCATAACTTAGGTTTAATGTATTCAGGTCAAA | **response regulator; putative**  Context:pXO1+[172308-173402] |
| Ba68 | CGGATCAGTTTTTACAATACTACTACCACTTCTTT |  |
| Ba69 | AATGATGCAATTAGTGCTTTACAGTCGAATCAACT | **s-layer protein**; (pxo1-90) |
| Ba70 | AAAGGACGCAAACGAAGGTGGGACTTTT | **spore germination protein xa**  Context:pXO1 |
| Ba71 | CGCATTCCTGCTGCATTTGGACAAACTTT |  |
| Ba72 | CCCAGTAATAAAAGGGATGATTTATCCAGCCGC | **spore germination protein xb**  Context:pXO1 |
| Ba73 | ACTCGTTATTTTTCTCATTCCAGATGTGCTACAAG |  |
| Ba74 | GATTTGTAGATGAAAAGAACCGTCATTATAACCGT | **thermonuclease family protein**; (pxo1-141) |
| Ba75 | ATGACAACTATTCAAGCAGGTAATGAAATGCACAA | **transcriptional regulator; arsr family**  (pxo1-138) |
| Ba76 | TTAGATATTCCTCAATCTACCGTATCTCAGCATTT |  |
| Ba77 | AGGTAATGAAATGCACAAAATCCCAGAGGCG |  |
| Ba78 | CTCGCTCTTGCTTAGATGATGTTCAGGAGTACA | **tyrosinerecombinase**  Context:pXO1 |
| Ba79 | TCTTCCGTATTAGCTACTCGCTCTTGCTTAGAT |  |
| Ba80 | TTCCTATTTCTATGCTAGTTGAAAAACCACCTCTG | **utp-glucose-1-phosphate uridylyltransferase** (pxo1-94) |
| Ba81 | GATTTTGCACTTCAGAGAGAAAGTCTAAAAGAAGA |  |
| Ba82 | TCGCCTTTGAATTTGATGGGAAAAGATATGATGTA |  |
| Ba83 | ATGTCTCAAGCAATTGATAGATGTTTATGAGGAAC |  |
